# Recent Progress in Advanced Nanobiological Materials for Energy and Environmental Applications

**DOI:** 10.3390/ma6125821

**Published:** 2013-12-11

**Authors:** Hyo-Jick Choi, Carlo D. Montemagno

**Affiliations:** National Institute for Nanotechnology and Department of Chemical and Materials Engineering, University of Alberta, Edmonton, AB T6G 2M9, Canada

**Keywords:** cellular life process, artificial organelle, polymersome, bioreactor, foam, water purification, biofuel, carbon dioxide sequestration

## Abstract

In this review, we briefly introduce our efforts to reconstruct cellular life processes by mimicking natural systems and the applications of these systems to energy and environmental problems. Functional units of *in vitro* cellular life processes are based on the fabrication of artificial organelles using protein-incorporated polymersomes and the creation of bioreactors. This concept of an artificial organelle originates from the first synthesis of poly(siloxane)-poly(alkyloxazoline) block copolymers three decades ago and the first demonstration of protein activity in the polymer membrane a decade ago. The increased value of biomimetic polymers results from many research efforts to find new applications such as functionally active membranes and a biochemical-producing polymersome. At the same time, foam research has advanced to the point that biomolecules can be efficiently produced in the aqueous channels of foam. Ongoing research includes replication of complex biological processes, such as an artificial Calvin cycle for application in biofuel and specialty chemical production, and carbon dioxide sequestration. We believe that the development of optimally designed biomimetic polymers and stable/biocompatible bioreactors would contribute to the realization of the benefits of biomimetic systems. Thus, this paper seeks to review previous research efforts, examine current knowledge/key technical parameters, and identify technical challenges ahead.

## 1. Introduction

**Figure 1:**
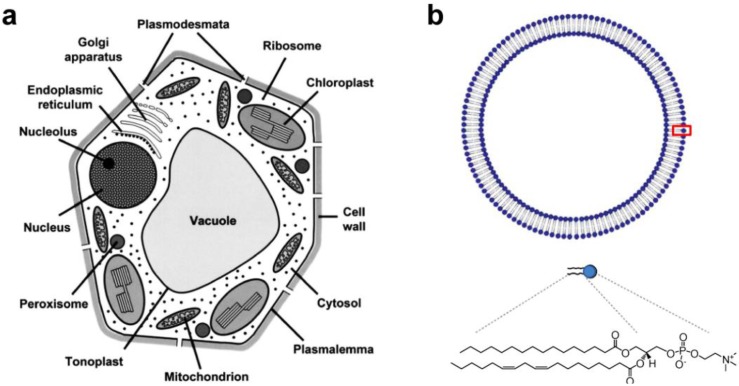
(**a**) Schematic diagram of a plant cell showing the main organelles and compartments (Reprinted with permission from [2]. Copyright 2007 Oxford University Press); (**b**) Schematic diagram of a lipid vesicle (liposome) made of phosphatidylcholine (PC).

Metabolic diversity in the cell is characterized by compartmentation of metabolism and function in cellular organelles, enabling the occurrence of multiple reactions in a separate but inter-dependent manner [1]. As shown in Figure 1a, plant cells are densely packed with small organelles such as vacuoles, chloroplasts, mitochondrion, endoplasmic reticulum, peroxisomes, *etc.* Each intracellular organelle is defined by a lipid bilayer membrane with embedded transport proteins/peptides that allow the passage of substances to maintain diverse cellular metabolisms. Cellular metabolisms are also controlled by enzymatic reactions, and in many cases driven by the biological energy source adenosine triphosphate (ATP), produced from mitochondrion and/or thylakoids inside a chloroplast. Therefore, from a structural point of view, the defining features of a cellular space are its membrane proteins and their compartmentation.

As envisioned in a previous report, biotic/abiotic hybrid technology utilizes biomolecular components as part of a device or applies their physical-biochemical principles in constructing nanobio devices [3]. Membrane-bound transport proteins in particular can be compared to biomolecular machines that function as unit operators. The “machines” can perform tasks that manifest filtering (aquaporin), pumping (bacteriorhodopsin), sensing (MscL), and energy transduction (F_O_F_1_-ATP synthase) functions. This makes integral membrane proteins well suited as design elements for application in engineering. Thus, lipid bilayers, shown in Figure 1b, with embedded proteins are thought to represent a platform, not only for mimicking subcellular structure, but for selectively replicating key aspects of metabolic processes. Integral membrane proteins partition into the liquid-ordered lipid phase and fold into an active conformation following the minimum free-energy pathway [4]. As a result, the stability and function of membrane proteins is closely associated with intrinsic properties of lipid membranes. Phospholipids comprise the majority of eukaryotic membranes and phosphatidylcholine (PC) is a major phospholipid class (>50%) (see Figure 1b, bottom, for chemical structure of PC with double bonds) [5,6]. Lipid composition and even thickness are very site-specific and vary significantly depending on the physiological role of the intracellular organelles [6]. The hydrophobic interaction accounts for the assembly of lipid molecules [7,8]. A dimensionless packing parameter (*v*/*a*·*l*, where *v*, *a*, and *l* are hydrocarbon volume, hydrocarbon/water interfacial area, and hydrocarbon chain length, respectively) used by Israelachvili *et al.*, accounts for the size and shape of lipid aggregates [9]. The physicochemical nature of lipids, however, is responsive to environmental factors (pH, ions, temperature, *etc.*) which can decrease the lifetime of liposomes due to hydrolytic and oxidative decomposition, as well as through the order-disorder phase transition [10,11]. One can expect that the overall lack of long-term stability and narrow tolerance to variation in physicochemical conditions make the lipid bilayer membranes found in cells unsuitable for the creation of engineering devices. Therefore, synthetic polymer membranes gain special attention because they have been demonstrated to preserve structural integrity of integral membrane proteins, and to create more stable membranes than lipids [12].

Early stage research has been focused on the demonstration of integral proteins’ activity in the biomimetic membrane and its application has been further extended to the creation of the so called “artificial organelle”, performing protein-specific cellular biochemical processes. Previous reviews present more general overviews on biomimetic polymersomes and/or their biomedical applications [13,14,15,16]. On the other hand, the primary goal of our research has been more directed toward solving engineering problems through the creation of new application models based on the biomimetic technology. As can be seen from the previous report, protein-incorporated polymersomes can be used to create nanobio devices (*i.e.*, energy sources for biomolecular machines, intervesicular/intravesicular communication, excitable vesicles) [3]. Unlike previous reports, this review focuses on the application of biomimetic polymer membranes to energy and environmental problems. There is no doubt that the search for new applications can become the basis for further investigation on biomimetic membranes, accelerate technology development, and provide the best way to gain more general attention.

This review is organized into five sections. We present the progress in biomimetic polymer membrane research as a mimic of lipid membrane, discuss the potential technical barriers for future applications, and introduce the two most challenging applications of biomimetic membrane technology (water purification and biofuel production) followed by future prospects and technical challenges: (a) development of artificial cellular membranes; (b) effects of polymer characteristics on the stability and function of proteins; and (c) advanced biological structure and function: applications of protein-incorporated polymer membranes. Since the scope of the paper is about the polymeric membranes compatible with integral membrane proteins, we do not attempt to touch on the general characteristics of block copolymers, nor structures formed by their self-assembly [17,18]. However, when the information on the biomimetic membrane alone is not sufficient, we seek out knowledge from the literature published on other types of polymers.

## 2. Development of Artificial Cellular Membrane

Key characteristics of biomimetic membranes include biocompatibility (unless mentioned otherwise, the biocompatibility used herein indicates the same meaning as compatibility with integral membrane proteins), stability in harsh environmental conditions, amphiphilicity, easy functionalization and size control, as well as ease of large scale production. These requirements are strict and may contain conflicts between our goals (e.g., mechanical stability of the membrane and stability of the embedded protein). As discussed below, herein lies our hope to find the potential of poly(dimethylsiloxane) (PDMS)-based block copolymers as an alternative to natural lipids.

### 2.1. Hydrophobic Backbone

The size of the hydrophobic segment determines both the morphology of the aggregates in aqueous solution (the hydrophobic fraction in total mass, *f*_hydrophobic_, of about 60%–70% forms polymersomes) and the mechanical characteristics of the membrane [19]. Mechanical stability of polymersome membranes increases with hydrophobic thickness and further by crosslinking polymers [20]. The elastic area modulus (*K*) of poly(ethylene oxide)-poly(dimethylsiloxane) (PEO-PDMS) graft polymer with 1.5 kDa of PDMS was reported to be ~93 mN/m with a critical area strain (α) of ~0.075 [21]. The *K* values are similar to or a bit less than those of liposomes (60–300), but the α values are higher than those of liposomes (<0.05) [22,23,24,25]. The *K* depends on the interfacial tension, *i.e.*, composition, of the membrane [26] and the bending modulus is proportional to *K*·*h*^2^, where *h* is membrane thickness [25]. Importantly, surface-modification or thickness control of the polymer can present a flexibility in controlling mechanical properties of the polymer membranes [20,21,27], including a method for the design of physically stable polymer membranes. On the other hand, considering that proteins fold mainly in the fluidic hydrophobic domain, the maintenance of membrane fluidity may be of equal importance for optimal activity of the proteins.

To date, PDMS has produced desirable performance as a hydrophobic backbone of biomimetic polymers as measured by protein function in the PDMS-based block copolymer membranes. The unique properties of PDMS, such as low intermolecular forces and flexibility, are correlated with easy rotation of the methyl groups around the (Si–O) bonds, leading to a low glass transition temperature (*T*_g_ = −123 °C [28]), low surface tension (15–22 mN·m^–1^ [29]), low solubility parameter (δ = 7.3 cal^1/2^·cm^−3/2^ [30]), high hydrophobicity, and chemical-physical-biological inertness [31]. Accordingly, when combined with a proper hydrophilic block, PDMS-based amphiphilic polymer can maintain good self-assembly properties in aqueous solution by maintaining membrane flexibility. The phase behavior of the poly(siloxane)-based polymers and their use in polymersome formation have been reported by many researchers [32,33,34,35], which can in turn be used to form polymersomes or protein-incorporated polymersomes.

### 2.2. Amphiphilic Block Copolymer

Synthesis of AB and ABA type amphiphilic block copolymers consisting of poly(oxazoline) (A: hydrophilic block) and poly(siloxane) (B: hydrophobic block) was first reported in the 1980s for applications in surfactants, stabilizers, and biomaterials [36,37]. The *T*_g_ of the resulting copolymer increases with increasing weight percentage of the poly(oxazoline) due to the high *T*_g_ of poly(2-ethyloxazoline (abbreviated as PEOX, *T*_g_ = 50 °C for 5 kDa)) [37]. This provided key methods for the synthesis of biomimetic block copolymers. However, the molecular weight of their polymers was outside the polymersome forming range. In 2000, Meier and his colleagues demonstrated biocompatibility of an amphiphilic block copolymer. OmpF (trimer: 110 kDa), a non-specific transport channel protein [38], was incorporated into the 10-nm-thick planar membrane made of ABA triblock copolymer (A: poly(2-methyloxazoline), abbreviated as PMOXA, B: PDMS; *M*_n_ = 9 kDa, *f*_hydrophobic_ = 60%) which maintained its passive transportation activity [39]. Further, the polymer showed a greater degree of control over morphology by the formation of polymersomes, simply by varying preparation conditions [40]. The transporting activities of other membrane proteins (e.g., aquaporin) were confirmed on the block copolymer membranes consisting of poly(siloxane) and poly(oxazoline) [41].

### 2.3. Characteristics of Biomimetic Block Copolymers

**Figure 2:**
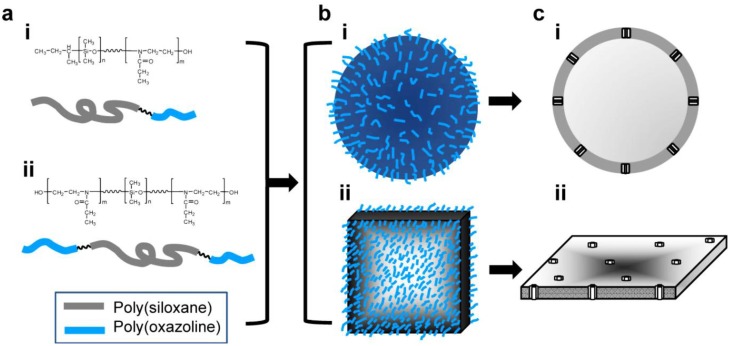
(**a**) Chemical structure of (**i**) poly(dimethylsiloxane)-block-poly(2-ethyloxazoline), PDMS-PEOX, AB diblock copolymer; and (**ii**) poly(2-ethyloxazoline)-block-poly(dimethylsiloxane)-block-poly(2-ethyloxazoline), PEOX-PDMS-PEOX, ABA triblock copolymer. Two representative structures of the biomimetic block copolymers: (**b**) schematic representation of (**i**) polymersomes and (**ii**) planar membranes; (**c**) cross-sectional view of the protein-embedded (**i**) polymersomes and (**ii**) planar membranes.

Figure 2a shows the chemical structure of synthesized AB (i) and ABA (ii) block copolymers (A: PEOX, B: PDMS). Upon hydration, the block copolymers spontaneously self-assemble to form polymersome (Figure 2b(i)) or planar membrane (Figure 2b(ii)) by exposing hydrophilic chains into the solution. The critical micelle concentration (CMC) of PDMS_500_-PEOX_2000_ diblock copolymer (*f*_hydrophobic_ = 20%) was determined to be 0.028 mM, significantly lower than that of sodium dodecyl sulfate (8.1 mM) [37]. Block copolymers meeting the *f*_hydrophobic_ criteria for polymersomes are expected to have a CMC value significantly lower than 0.028 mM because of the lower free energy penalty to form micelles with the increase and decrease of hydrophobic and hydrophilic blocks, respectively [42]. As shown in Figure 2c, membrane proteins span the hydrophobic domain of the polymersome wall (i) and planar membrane (ii).

**Figure 3:**
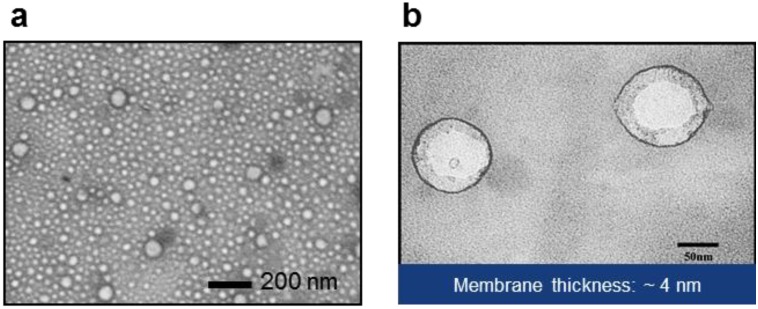
(**a**) Low- and (**b**) high-magnification TEM images of polymersomes (PEOX-PDMS-PEOX), with wall thickness of about 4 nm. Image in (**b**) adapted with permission from [43] (Copyright 2007 IEEE).

Transmission electron microscopy images of polymersomes are shown in Figure 3 (a: low-magnification, b: high-magnification). Mean thickness of the hydrophobic core of the wall was around 4 nm, which is similar to the size of integral membrane protein [43]. However, the thickness of free-standing planar membrane was measured to be about 9 nm by electrochemical impedance spectroscopy [44]. The observed thickness difference between polymersomes and planar membranes may be attributed to shape-dependent conformation change of the polymer molecules or errors in thickness estimation based on capacitance measurement. In discussing the thickness of the hydrophobic layer of polymersomes, critical factors are thought to be: molecular weight of the hydrophobic block, conformation of the copolymer, and polydispersity. Battaglia and Ryan derived the thickness of the hydrophobic layer of block copolymer vesicles using a power law: $t\;\approx \;a\chi^{1/6}N^{2/3}\propto \;M_{w}^{2/3}\;$, where *a*, *χ*, and *N* are the chain unit length, the Flory-Huggins parameter, and the number of hydrophobic units, respectively [45], and *M_w_* indicates the molecular weight of the hydrophobic block of the polymer. As a result, block copolymers composed of poly(ethylene oxide) (PEO) and poly(butylene oxide) (PBO) with *N*~70 exhibited membranes about 4.5 nm thick. In the same report, it was found that AB, ABA, and BAB block copolymers formed polymersomes with interdigitated membrane structure and follow the same power rule. On the other hand, Salva *et al.* [46] recently reported that PEO-PDMS (graft copolymer, *M_v_*: 3 kDa, thickness: ~5 nm) and PEO-PDMS-PEO (triblock copolymer, *M*_n (PDMS)_: 5 kDa, thickness: ~11 nm) probably form polymersomes with a bilayer and a monolayer, respectively, with different hydrophobic thickness. While accepting the difference in the chemical structure of polymers, these reports indicate that the conformation of the block copolymer in the membrane play a critical role in determining the hydrophobic thickness of the polymersomes. For an example, flexible polymers may prefer an interdigitated structure to a bilayer, which accounts for higher membrane thickness of poly(ethylene oxide)-polybutadiene (PEO-PBD) than that of PEO-PBO [26,45]. This further suggests the importance of the chemical characteristics in determining membrane thickness. Another possible factor affecting conformation might be an unequal size of end blocks for ABA and BAB due to different polymerization reaction rates at the ends of central blocks. That is, ABA triblock copolymers with a size unbalance between two hydrophilic blocks, *i.e.*, ABAʹ, may self-assemble into a configuration different from a balanced triblock copolymer. As summarized in the previous report by Lo Presti *et al.* [47], block copolymers can be assembled into membranes with various conformations depending on their structure. Further, another factor to consider is polydispersity of polymers; unlike natural lipids, synthetic polymers possess chain length differences, *i.e.*, polydispersity. The non-uniformity of synthetic polymers may result in irregularity of the membrane thickness inside a polymersome or between polymersomes. The difference in hydrophobic length between protein and polymer membrane could be important to the incorporation and stability of proteins. The level of hydrophobic mismatching affects the conformation and activity of the incorporated proteins. However, those polymer size effects can be predicted for the most part, as discussed below, from knowledge learned from lipids, due to limited reports on the evaluation of biomimetic polymer membranes.

**Figure 4:**
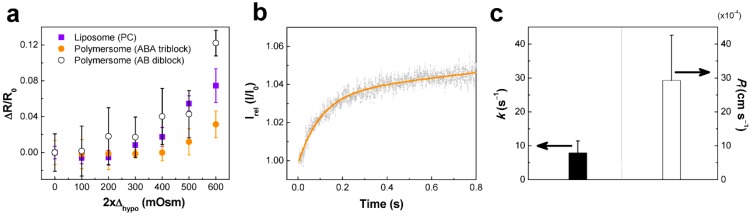
Membrane characteristics of polymersomes. (**a**) Relative swelling of PC-liposomes, ABA polymersomes, and AB polymersomes at hypo-osmotic stresses; (**b**) Hyperosmotic stress-induced stopped-flow light scattering of polymersomes at 300 mOsm of hyper-osmotic stress and 4 °C, and (**c**) their corresponding rate constant (*k* [s^−1^]) and osmotic water permeability coefficient (*P*_f_ [cm·s^−1^]). The hypo-osmotic stress-induced size change was characterized using dynamic light scattering at 4 °C and the relative size change (∆*R*/*R*_0_ = (*R* – *R*_0_)/*R*_0_, *R* and *R*_0_ are the radii at hypo-osmotic (∆_hypo_) and iso-osmotic conditions, respectively) was plotted against 2∆_hypo_·*k* was measured by curve fitting of light scattering spectra shown in (**b**) using the equation *I* = a + b∙e^–*k*∙*t*^, where a and b are constants. *P*_f_ was calculated by *P*_f_ = *k*∙*V*_0_/*A*∙*V*_w_∙∆, where *k =* rate constant, *V*_0_ = initial volume, *A =* surface area, *V*_w_ = molar volume of water, and ∆ = osmotic gradient [48]. Osmolarity was controlled by varying NaCl concentrations.

Polymersomes are inevitably exposed to osmotic stress due to their hollow interior. In this paper, we focus more on thin-walled biomimetic polymersomes, thus the general osmotic behavior of polymersomes is not summarized here. Physical properties of biomimetic polymersomes can be understood in comparison to those of liposomes in response to osmotic stress. When exposed to hypotonic osmotic stress, polymersomes and PC-liposomes exhibited typical osmotic swelling response as indicated by the increase of relative size (∆*R*/*R*_0_, *R*_0_: radius in iso-osmotic condition) (Figure 4a). It is noted that polymersomes made of triblock copolymer (ABA, PEOX-PDMS-PEOX) exhibited the lowest level of swelling, and there was no significant difference between polymersomes made of diblock copolymer (AB, PEOX-PDMS) and PC-liposomes. Based on an inverse relationship between ∆R/R_0_ and elastic membrane compressibility modulus, *K* [22], ABA-polymersomes are estimated to exhibit the highest level of resistance to deformation (*K*_polymersome (ABA)_ >*K*_polymersome (AB)_, *K*_PC-liposome_). Higher resistance of the triblock copolymer against hypo-osmotic swelling is consistent with the previous report by Salva *et al.* [46] on the PEO-PDMS/PEO-PDMS-PEO polymers.

Hypertonic osmotic shrinkage of polymersomes (ABA) was investigated using stopped-flow light scattering at hyperosmotic stress difference of 300 milliosmolarities (mOsm) at 4 °C. As shown in Figure 4b, the light scattering curve of polymersomes exhibited a rapid increase in intensity at the initial stage, followed by a gradual saturation. The rate constant (*k*) and osmotic water permeability (*P*_f_) values of polymersomes were measured to be ~8 s^−1^ and ~3 × 10^−3^ cm·s^−1^ (Figure 4c). It is estimated that osmotic water permeability of the biomimetic polymer membrane can significantly vary depending on polymer properties (*i.e.*, polymer length, polydispersity, chemical properties), membrane packing density (*i.e.*, membrane defects), and aqueous media (*i.e.*, pH, buffer composition). This explains the difference in osmotic water permeability compared with similar polymersomes [41,49]. In general, the membrane permeability decreases with increasing the hydrophobic thickness of the polymersome wall [47,50]. It must be mentioned that the biomimetic polymer membrane did not allow passage of osmolytes, as evidenced by the lack of osmotic dissipation, which is typically displayed as a gradual decrease in scattered light intensity. This is an important characteristic in the application of polymer membranes because membranes displaying leakage to substances (ions or molecules) cannot be used in the creation of water purification membranes, and cannot generate a concentration difference of biological agents across the membrane used to drive a variety of different biochemical reactions. Therefore, it will be critical to modulate the permeability of the biomimetic polymer membranes to meet their application requirements.

## 3. Effects of Polymer Characteristics on the Stability and Function of Proteins

The maintenance of active protein conformation in the polymer membrane is a key factor in obtaining maximum levels of protein activity. Functionality of protein-incorporated polymer membranes is strongly affected by the protein-membrane and protein-protein interactions caused by hydrophobic mismatch, the mechanical property of the polymer membrane, and the characteristics of the proteins. It is anticipated that such membrane-associated problems are more likely to occur in synthetic polymeric membranes than in natural lipid membranes. Therefore, understanding the protein stability in the natural membrane would provide insightful guidelines on the design of the biomimetic cell membranes. Studies on lipid membranes have been documented in many reviews [51,52,53,54,55,56,57,58,59,60]. In this paper, we do not summarize previous reviews on lipids, but hope to utilize useful parameters learned from the lipid bilayer-protein systems to better predict the protein stability in the polymer membranes.

**Figure 5:**
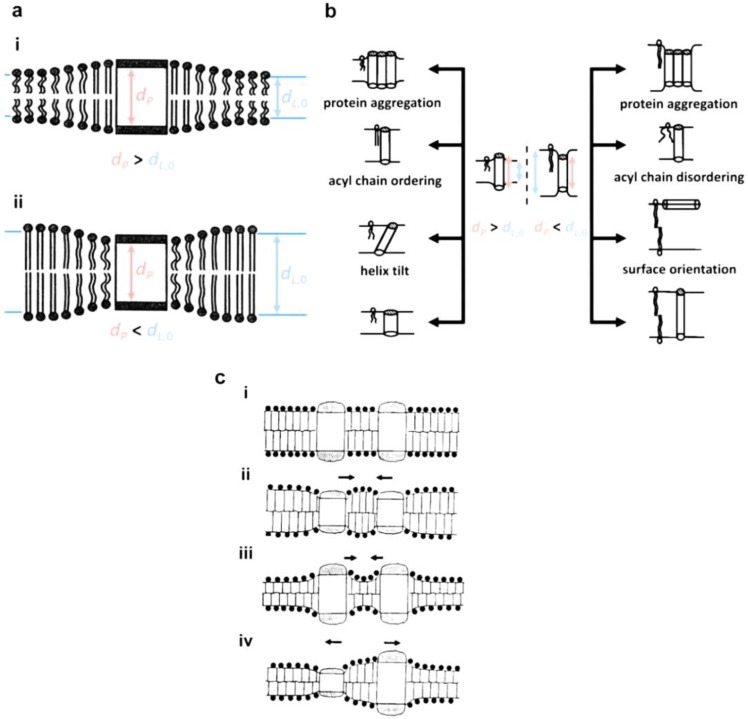

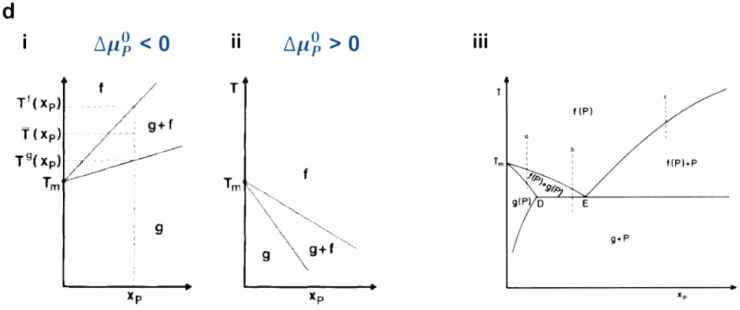
(**a**) Schematic illustration of hydrophobic mismatching between protein and bilayer (adapted with permission from [79]. Copyright 1999 Elsevier.); (**b**) Possible consequences of hydrophobic mismatching (adapted with permission from [54]. Copyright 1998 Elsevier.); (**c**) Protein-protein interactions induced by hydrophobic mismatch (reprinted with permission from [80]. Copyright 1995 The Royal Society of Chemistry.); (**d**) Schematic phase diagrams for a binary lipid-protein mixture at low protein concentration regime ((i): $∆\mu_{p}^{\text{o}}\;<\;0$), (ii): $∆\mu_{p}^{\text{o}}\;>\;0$) reprinted with permission from [81]. Copyright 1988 Springer Science and Business Media.), and (iii) at high concentration regime with eutectic, reprinted with permission from [82]. (Copyright 1984 Elsevier). (f: fluid phase, g: gel phase.)

Hydrophobic mismatch is the difference in hydrophobic length between amphiphilic molecules (*d_L_*_,0_) and proteins (*d_P_*_,0_). It is a primary determinant of the function and stability of the integral membrane proteins. In general, *d_P_* = *d_P_*_,0_ is a good assumption because compressibility of proteins is two to three orders of magnitude higher than that of lipids [60]. Two possible hydrophobic mismatches are shown in Figure 5a: (i) *d_P_* > *d_L_*_,0_; (ii) *d_P_* < *d_L_*_,0_. As shown in the schematic, size differences may induce stretching or compression of both membrane and protein to minimize the excess free energy [53]:

(1) $$∆G=\frac{2\pi b}{{\left(d_{L,0}^{f}-d_{L,0}^{g}\right)}^{2}}\left(\frac{\rho_{P}}{\pi \xi_{L}}+1\right){\left(d_{P}-d_{L}\right)}^{2}$$

where *b*, $\rho_{P}$, and $\xi_{L}$ denote a constant associated with an elastic energy, the circumference of the protein, and the coherence length of the lipid-bilayer fluctuations, respectively. $d_{L,0}^{f}$, $d_{L,0}^{g}$, $d_{P}$, and $d_{L}$ represent the lipid bilayer thickness in the fluid phase, lipid bilayer thickness in the gel phase, equilibrium protein thickness, and equilibrium lipid membrane thickness. As a result, hydrophobic mismatch induces the lipid bilayer to change its hydrophobic thickness by extension (Figure 5a(i)) or compression (Figure 5a(ii)) according to the function:

(2) $$d_{L}\left(r\right)=d_{L,0}+\left(d_{P}-d_{L,0}\right)\cdot e^{-r/\xi_{P}}$$

where *r* is the distance from the protein and $\xi_{P}$ is the coherence length of the protein perturbation [61]. As shown in Figure 5b, the structure, orientation, and length of the membrane, as well as the conformation, tilt, orientation, aggregation, and length of protein/peptides, responds to hydrophobic mismatch in a way to alleviate the energy cost. As a consequence, the stability and function of integral membrane proteins strongly depends on the level of deformation of both the membrane and the protein, *i.e.*, extent of hydrophobic mismatch. This explanation can be supported by experimental reports on the chain length-dependent activity change of cytochrome c oxidase [62], F_O_F_1_-ATP synthase [62], Na^+^, K^+^-ATPase [63], Ca^2+^-ATPase [64], gramicidin [65], MscL [66], rhodopsin [67], bacteriorhodopsin [68], lactose permease [69], and melibiose permease [70]. Although there are no reports on the effects of polymer chain length on the activity of proteins, it is expected that hydrophobic mismatch would play a critical role in determining functional activity of the protein-embedded polymer membranes (polymersomes, planar membranes). This further indicates that, without doubt, synthesizing biomimetic block copolymers with a minimum hydrophobic mismatch is of the utmost importance [71]. Fortunately, unlike natural materials, a rich variety of polymers with different functional groups may be able to provide a solution to technical hurdles (e.g., hydrophobic mismatch). Examples are the report of Meier’s group on protein activity in a thick polymer membrane (~10 nm) [72,73] and a report on the successful incorporation of dopamine receptor D2 into a thick planar membrane [74]. Under simplified conditions, Pata and Dan predicted that the concentration of proteins decreases with increasing hydrophobic mismatch, and perturbation decay length of the polymer membrane is relatively longer than that of lipid bilayers [75]. In spite of the need for further investigation to find the tolerance limit of the polymer, it is promising to see the possibility of overcoming the size difference issue using flexible polymers.

Polydispersity of polymers may also have a significant effect on membrane proteins. Due to the interactions described above, in a membrane with a distribution of polymer chain lengths, polymers with minimal hydrophobic mismatch would preferentially bind to proteins. This amphiphile species/size-dependent, selective protein interaction was predicted by Sperotto and Mouritsen [76,77,78] and experimentally demonstrated for bacteriorhodopsin [68] and *E. coli* lactose permease [69]. The formation of two different phases (protein-rich and poor phases, or amphiphile-rich and poor phases) may influence not only the stability of membranes, but the activity of the proteins. At a given temperature, compositional changes of lipids in a binary lipid system would result in a phase transformation of the lipid, inducing protein aggregation due to solubility difference in the gel and fluid phases.

The protein-rich phase would have an increased possibility of protein-to-protein interaction, as predicted by the mattress model (Figure 5c) [81]. In the case of polydisperse polymers, the membranes could be in the form of a mixture of glass and liquid phases or a combination of phases: one phase rich in long-chain polymers and the other rich in short-chain polymers. Thus, membrane proteins would preferentially be located in the liquid phase and/or in the phase with less hydrophobic mismatch [75]. Although the chain length-dependent phase diagram is not currently reported, it can be used to improve predictions optimal polymer conditions for protein incorporation. To date, an unsolved question is whether the maintenance of protein activity in thicker polymer membranes is associated with the flexibility of the polymer or the selective incorporation of proteins into thin membrane areas. Therefore, from a practical application point of view, the effect of polydispersity on the insertion and activity of membrane proteins would provide important information for future biomimetic membrane research.

According to the mattress model, lipid-protein interactions due to hydrophobic mismatch are known to induce both change in the phase transition temperature of the lipid membrane and phase separation [81]. As shown in Figure 5d(i) and (ii), the midpoint transition temperature ($\bar{T}\left(x_{P}\right)$) varies as a function of protein concentration under dilute conditions:

(3) $$\bar{T}\left(x_{P}\right)≅T_{m}+\frac{R{T_{m}}^{2}}{∆H_{L}}x_{P}\sin\text{h}\left(\frac{\rho_{P}\cdot \Gamma }{RT_{m}}\right)$$

where $T_{m}$, $∆H_{L}$, Γ, and $x_{P}$ represent the phase transition temperature of pure lipid, phase transition enthalpy, a function of both energy and hydrophobic thickness parameters, and protein concentration. The increase or decrease of the phase transition temperature with protein concentration depends on the sign of chemical potential at the standard state, and the phase transition temperature of the membrane can be expected to increase or decrease at $\mu_{P}^{0,g}<\mu_{P}^{0,f}$ (a tendency of proteins to favor the gel phase, Figure 5d(i)) and $\mu_{P}^{0,g}>\mu_{P}^{0,f}$ (a tendency of proteins to favor the fluid phase, Figure 5d(ii)), respectively. However, at high protein concentrations, protein aggregation can be observed with a gel or fluid phase, as can be seen in the phase diagram of Figure 5d(iii). Protein aggregation is related to the protein-protein and protein-membrane interactions shown in Figure 5c. Theses interactions would be critical in determining maximum protein incorporation limit for optimal functionality of protein-incorporated polymer membranes, *i.e.*, a protein level in the membrane that does not hamper the stability/activity of proteins and membranes [80,83]. In terms of protein aggregation, extramembranous domain interactions might also be an important parameter [57,84]. Contacting hydrophobic domains of proteins would be interfered with by the presence of large extramembranous domains, as exemplified by Ca^2+^-ATPase [57,84]. This indicates that the incorporation of membrane proteins into the polymer membranes would influence the *T*_g_ of the polymer and increase protein-protein interactions, which may affect the activity of membrane bound proteins.

Due to lack of information, our prediction about the effect of hydrophobic mismatch on transmembrane protein-polymer systems is based mostly on the current understanding of lipid-protein systems. It is expected that the difference in fundamental material properties might cause a difference in the binary phase diagram between biomimetic polymers and proteins. However, it is reasonable to assume that the principle of protein insertion and folding would be the same regardless of the kinds of amphiphiles (biomimetic polymer and lipids). Thus, proteins respond to any favorable/unfavorable conditions during and after insertion into the membrane by means of stability and functional activity change. As such, there is no doubt about the negative effects of hydrophobic mismatch on the stability/activity of proteins in the biomimetic polymer membrane. However, the remaining questions are, as discussed above, how much tolerance to hydrophobic mismatch is allowed and how flexibility and polydispersity of the biomimetic polymer affect the protein-incorporated polymer membranes. Experimental and theoretical investigations on these will contribute to the selection and design of optimal biomimetic polymers.

## 4. Advanced Biological Structure and Function: Applications of Protein-Incorporated Polymer Membranes

### 4.1. Protein-Incorporated Biomimetic Polymer Membrane: Artificial Organelle

**Figure 6:**
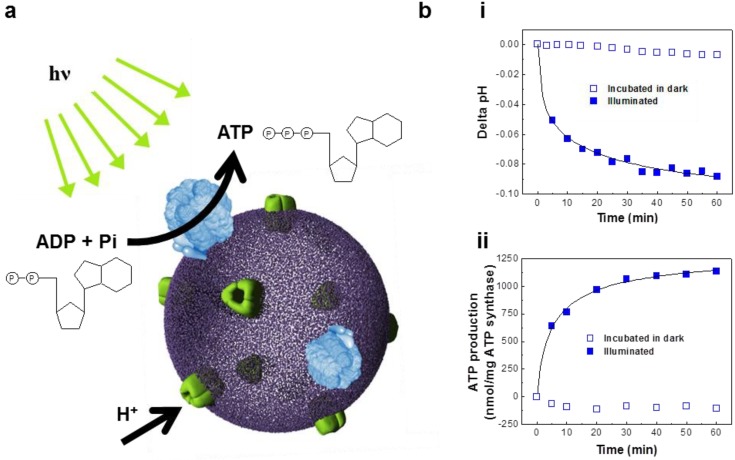
(**a**) Schematic representation of ATP-producing polymersome (BR-ATP synthase-polymersome) (adapted with permission from [43]. Copyright 2007 IEEE.). (**b**) (**i**) Intravesicular pH change as a measure of proton pumping activity of BR-polymersomes and (**ii**) photosynthetic ATP production in the BR-ATP synthase-polymersomes. (adapted with permission from [85]. Copyright 2005 American Chemical Society.)

*In vitro* biochemical synthesis is required to reconstruct cellular functions. Protein-incorporated polymersomes can act as an artificial organelle, performing complex biological processes *in vitro* [85,86,87,88,89]. As part of our efforts to engineer complex life process, we have demonstrated ATP synthesis from bacteriorhodopsin and F_O_F_1_-ATP synthase reconstituted polymersomes (abbreviated as BR-ATP synthase-polymersomes) (Figure 6a) [85].

This study reported the induction of biochemical reactions through the orchestration of two protein species, stimulated by illumination. To demonstrate ATP synthesis activity, a well-known chemiosmosis was adopted [90]. BR pumps protons to create a pH gradient across the membrane with the use of light (Figure 6b(i)) [91,92]. This electrochemical proton gradient is coupled to the rotational catalytic activity of F_O_F_1_-ATP synthase (Figure 6b(ii)) for ATP synthesis [93,94]. Therefore, the present findings imply that BR and ATP synthase proteins maintain their functional conformations, *i.e.*, rotational motion of the membrane bound F_O_ complex and the light-activated proton pumping activity of BR, in the biomimetic polymersomes. Despite the need for optimization of membrane thickness and protein-protein/protein-lipid ratio, this finding can be further extended to the engineering multiple-polymersome level life process with a higher architectural complexity.

### 4.2. Reverse Osmosis Water Purification Membrane

#### 4.2.1. Background

**Figure 7:**
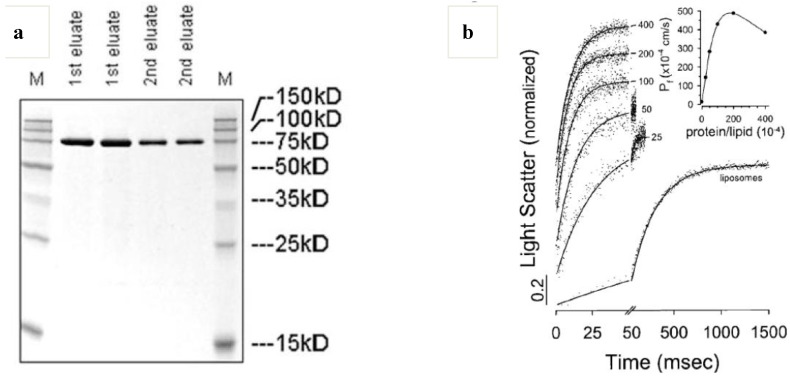
Aquaporin Z-incorporated vesicles. (**a**) Sodium dodecyl sulfate-polyacrylamide gel electrophoresis (SDS-PAGE) of purified aquaporin Z (M: marker) and (**b**) stopped-flow light scattering and osmotic water permeability coefficient of aquaporin Z-incorporated liposomes in response to hyperosmotic stress (reprinted with permission from [48]. Copyright 1999 Elsevier).

Aquaporins (Aqps) are membrane water channels, playing important roles in regulating water transport in cells and thus contributing to the water homeostasis of organisms [95]. From a practical application point of view, *E. coli* aquaporin-Z (AqpZ) with a histidine-tag has advantages due to large-scale protein production and single-step purification (Ni-NTA or Talon resin) [48]. AqpZ forms a tetramer (70–80 kDa, see Figure 7a) which can transport water across the membrane in the presence of an osmotic gradient. It is noted that osmotic water permeability coefficient (*P*_f_) increases with increasing protein content, and decreases with a protein-to-lipid weight ratio >0.02, possibly through protein-to-protein interaction discussed in Section 3 (Figure 7b). AqpZ has been reported to selectively transport only pure water molecules across cellular membranes with a high water permeability (*P* ≥ 10^−13^ cm^3^·monomer^−1^·s^−1^) and a low Arrhenius activation energy (*E*_a_ = 3.7 kcal·mol^−1^) [48]. This corresponds to a water transport rate of about 3 × 10^9^ water molecules/monomer/s. This exceptionally high water transport capability and sharp water selectivity of AqpZ make AqpZ-embedded polymer membranes (polymersomes, planar membranes) an ideal tool in developing reverse osmosis (RO) membranes with a low energy cost.

#### 4.2.2. Mechanism and Design Parameters

**Figure 8:**
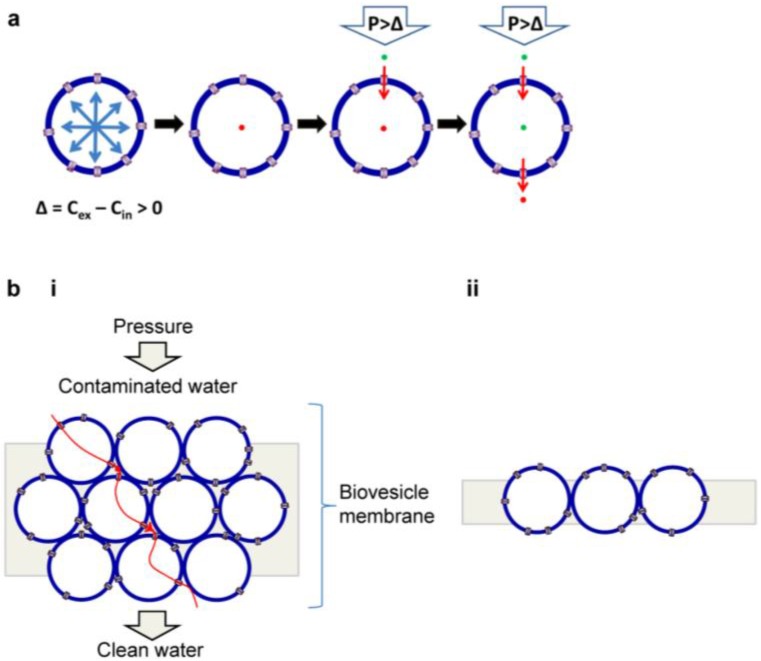
Schematic drawings representing our hypothesis of a reverse osmosis water purification membrane using aquaporin-incorporated vesicles. (**a**) Principle of reverse osmosis in a single vesicle and (**b**) schematic diagram of a membrane made with (**i**) multi-layer stacking and (**ii**) single-layer stacking of aquaporin-vesicles.

AqpZ in polymersomes can be used as an active water purification unit, as shown in the schematic diagram of Figure 8a. When exposed to hypertonic stress (∆ = *C*_ex_ – *C*_in_ > 0), AqpZ-polymersomes shrink to increase membrane packing density and remain in their spherical shape until the membrane can resist the osmotic stress. The hypothesis is based on the assumptions that: (1) mechanically stabilized AqpZ-polymersomes do not respond to osmotic gradients; (2) application of pressure (*P* > ∆) induces an influx of water molecules at the high-pressure side, and (3) AqpZ-polymersomes maintain a steady-state and thus input equals output (Figure 8a). The essential part of this idea is to use robust polymersomes that are irresponsive to both osmotic stress and applied pressure maintain functional activity of the embedded AqpZ proteins. This can be achieved by attaching polymersomes firmly to the surrounding matrix materials with the proper choice of cross-linking method and/or by the surface cross-linking of polymersomes. If polymersomes are sensitive to osmotic stress, they would shrink and experience significant morphological deformation upon continuous exposure to hypertonic osmotic stress from contaminated water or saline solution. This would result in the increase of interstitial spacing (increased leakage), the formation of membrane defects, activity decrease of proteins due to compression and membrane deformation, and failure to induce reverse osmosis due to efflux of water from the internal vesicular volume. Polymersomes have the advantage of easy membrane fabrication over planar membranes (discussed below). However, another important parameter to consider is packing density due to the spherical shape of polymersomes [96]. Theoretically, the maximum packing density of the close-packed polymersomes of the same size is 74%. Thus, even the most tightly packed membranes made with AqpZ-polymersomes would have 26% interstices, resulting in a decrease of rejection rate due to the occurrences of leakage path for contaminated water, salt precipitation, and pressure drop. Therefore, to increase membrane performance, it is critical to seal the interstices between AqpZ-polymersomes with filling materials or by embedding AqpZ-polymersomes into a leak-free coating. AqpZ-polymersome membrane can be composed of stacked multiple layers (Figure 8b(i)) or a single layer (Figure 8b(ii)).

**Figure 9:**
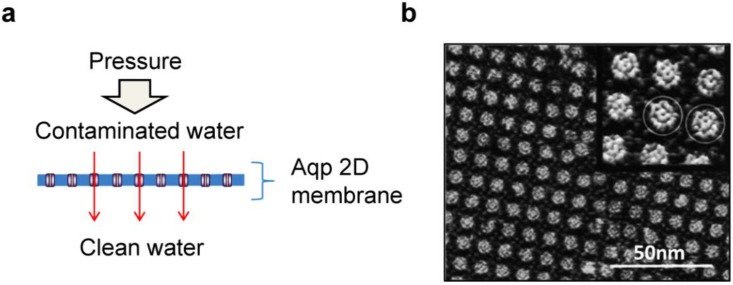
Reverse osmosis water purification membrane using aquaporin-incorporated planar membranes. **(a)** Schematic diagram of the reverse osmosis water purification membrane made with aquaporin-embedded 2-dimensional planar membrane; **(b)** AFM topography of AqpZ crystal (adapted with permission from [101]. Copyright 1999 Macmillan Publishers Ltd.).

AqpZ-incorporated 2D membranes coated on a substrate (Figure 9a) may be advantageous in terms of purification efficiency and energy cost, but bear significant drawbacks such as limited scale-up, and weakness to mechanical deformation and/or applied pressure, and difficulty in forming defect-free membranes. Vesicles can also be transformed into planar membranes under some process conditions [97,98,99,100]. To estimate the maximum performance of a 2D AqpZ-membrane, it is assumed that (1) the biomimetic membrane is a monolayer of AqpZ-incorporated planar membrane; (2) AqpZ is incorporated into the membrane with an optimal permeability and a maximum packing density as in two dimensional (2D) AqpZ crystal, and (3) the supporting substrate membrane does not affect the transporting behavior of water molecules (*i.e.*, no resistance to water movement). 2D AqpZ crystal is reported to have a tetragonal structure (*a* = *b* = 95 Å, *c* = 57 Å) with a p42_1_2 symmetry (Figure 9b) [101]; the maximum planar protein packing density is estimated to be ~1 × 10^16^ tetramer m^−2^. Under these simplified conditions, water flux of AqpZ-2D membrane is calculated to be 4.4 L/m^2^·s, two to three orders of magnitude higher than commercially available RO membranes [102,103]. Although this calculation was performed by ignoring both the maximum protein content in the polymer membrane and protein-protein/protein-membrane interactions, there is no doubt about the potential applicability of AqpZ-incorporated polymer membranes as an active component of RO membranes.

#### 4.2.3. Status

Since the first introduction of the concept of RO membranes using aquaporin-incorporated biomimetic membranes in 2003 [104], water transporting activity of AqpZ in the biomimetic polymer membrane was extensively investigated by Applied Biomimetic (Nordborg, Denmark) and Aquaporin A/S (Copenhagen, Denmark). Meier’s group tested the effects of polymer types on the orientation of Aqp-0 in 2004 [105] and the same group published the functionality of AqpZ-incorporated polymersomes in 2007 [41]. In the report, AqpZ-incorporated polymersomes maintained a similar level of water permeability to that in the liposomes. Because aquaporin-incorporated membrane should be mounted on a support, many research efforts were focused thereafter on the development of support membranes at the initial stage. Briefly, Aqp-incorporated planar lipid membranes on the hydrophilic surface or around a hydrophobic porous support were proposed as an active component of the water purification device, fabricated using Langmuir-Blodgett method, vesicle fusion method, and spin-coating method [106]. The CO_2_-laser ablation method was proven to provide advantages in making homogeneous apertures on hydrophobic surface as a support of lipid/polymer planar membranes [107,108,109]. However, mechanical stability of the formed planar membranes against applied pressure was not tested for application in reverse osmosis water purification membranes. In 2011, the same group addressed these stability issues and fabricated a black lipid membrane on a composite hydrogel casted across a multi-aperture partition [110]. While planar membranes on the hydrogel support survived up to 1 h longer than free-standing membrane, it is far from the stability level required for water purification membranes. Importantly, pressure-dependent stability of the membrane was not investigated in the report. As a different approach, it is notable that Kaufman *et al.* fabricated supported lipid membranes on a nanofiltration membrane, which were stable against a pressure of 10 bar [111]. In 2012, Li *et al.* [112] prepared AqpZ-embedded planar membranes on NF-270, but failed to detect functionality of the membrane, presumably due to the aggregation of liposomes or proteins on the substrate. Zhong *et al.* [49] reported the first successful measurement of RO membrane performance using AqpZ-incorporated biomimetic polymer membranes formed on the surface-modified cellulose acetate membranes. In the report, 34 L/(m^2^ h bar) of water permeability and 33% of NaCl rejection at 200 ppm NaCl were measured from the membranes. Although the performance was significantly below the level for practical applications, it is a meaningful result as the first demonstration of proof-of-concept. Following this, aquaporin-incorporated liposomes were embedded into the aromatic polyamide thin films by adopting interfacial polymerization of a polyfunctional amine such as m-phenylenediamine (MPD) with a polyfunctional acid chloride such as tri-mesoyl chloride (TMC) on a polysulfone support [113]. Such a membrane exhibited 4 L/(m^2^ h bar) of water permeability and 97% of NaCl rejection at 10 mM NaCl and 5 bar. Furthermore, the membrane was demonstrated to have good mechanical stability up to 10 bar. The improvement in the salt rejection and decrease in the permeability compared to the membrane reported by Zhong *et al.* [49] can be explained by the reduced membrane defects/ionic leakage paths due to the use of both ionic leakage-proof materials and mechanically stable supports, in addition to the major salt rejection through polyamide membranes.

#### 4.2.4. Prospects

High water selectivity and water permeability confer benefits to Aqps as an active element of RO water purification membranes. Following this concept, two general approaches have been used to purify water through reverse osmosis: Aqp-incorporated planar membranes or AqpZ-polymersomes. Although the idea of the Aqp-membrane has been successfully demonstrated, its unique advantage over conventional RO membranes has not been prominent due to several seemingly incompatible technical requirements. First, the increase in the volume fraction of Aqp-incorporated polymer membranes would increase the contribution of Aqp activity to the total performance of the water purification membranes. However, it would also increase both weakness of the membrane and ionic leakage, resulting in the decrease of salt rejection. However, the use of sealing materials to fill interstitial space between AqpZ-membranes such as embedding Aqp-polymer membranes into the matrix of seal coat, can result in higher salt rejection by reducing ionic leakage but decrease water permeability due to reduced pressure. In this case, the properties of the sealing material would dominate the performance of the RO membranes. Second, the increase in the mechanical strength of the biomimetic membrane might decrease the stability/activity of Aqps. That is, a flexible membrane decreases mechanical stability, but might be helpful for maintaining functional protein conformation. These two issues are associated with the use of protein-incorporated biomimetic membranes and have not been investigated in detail. Therefore, we estimate that, other than membrane fabrication and process parameters, future research should be directed toward developing methods to increase both protein stability and membrane stability and to prevent both ionic leakage and pressure drop, to maximize both salt rejection and water permeability. Such goals can be achieved by both the functionality increase of individual membrane components and the introduction of new membrane concepts.

### 4.3. Foam as an *in-Vitro* Bioreactor

#### 4.3.1. Background

**Figure 10:**
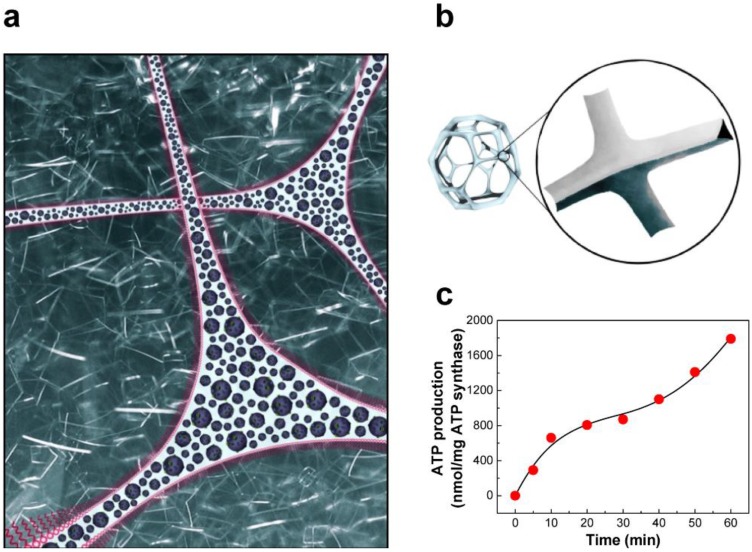
(**a**) Foam architecture as a platform for a bioreactor; (**b**) Plateau border of foam, aqueous channels formed at the junction of adjacent bubbles; (**c**) ATP synthesis by BR-ATP synthase-polymersomes in the aqueous channels of Tween-20 foam. (adapted with permission from [114]. Copyright 2006 IOP Publishing)

For the engineering of a cellular life process *in vitro*, it is crucial to create an environment where artificial organelles can be housed to perform programmed biochemical reactions through the exchange of substances between polymersomes. That is, artificial organelles should distribute densely within a small space that mimics functional cellular architecture, enhancing reaction kinetics and replicating cellular metabolism. With this in mind, we first proposed the potential applicability of foam as a bioreactor in 2006 (Figure 10a) [114]. To test proof-of-concept, BR-ATP synthase-polymersomes were encapsulated in the aqueous channels of Tween-20 foam (Figure 10b) and their ATP synthesis activity was confirmed, as shown in Figure 10c. The main drive behind this research is the belief that micro- or nano-sized channels may immobilize artificial organelles and provide an environment with a locally high concentration of biomolecules to the populated organelles, similar to a sub-cellular structure. Thus, this foam-based bioreactor is predicted to have a great advantage in living systems which require a high density of organelles within a small space (e.g., microalgae growth for biofuel production). Ultimately, owing to low cost and ease of process, aqueous channels of foam can be a platform for replicating sub-cellular environment.

#### 4.3.2. Mechanism and Design Parameters

Four representative elements must be satisfied in order to apply foams to a bioreactor: (i) foamability; (ii) foam stability; (iii) biocompatibility with embedded organelles; and (iv) scalability/cost-effectiveness. Foamability represents the ability of the foaming solution to convert to foam and is measured by the initial foam height as tested by the Ross-Miles method [115]. According to the Gibbs-Marangoni effect, the foaming process is strongly related to the dynamic surface tension, which can be expressed by the following empirical relationship [116]:

(4) $$\gamma_{t}-\gamma_{m}=\frac{\gamma_{0}-\gamma_{m}}{1+{\left(t/t^{*}\right)}^{n}}$$

where γ*_t_*, γ*_m_*, and γ_0_ indicate surface tension at time *t*, at meso-equilibrium, and of the pure aqueous solution. t* is the time for the surface tension to reach one half of the difference between γ_0_ and γ*_m_*: *n* is a constant related to the hydrophobicity of a surfactant. Since the rate of surface tension change is related to the Marangoni elasticity, a dynamic surface tension gradient at time t*, $n\left(\gamma_{0}-\gamma_{m}\right)/t^{*}$, can be interpreted as an indicator of the maximum Marangoni effect and the foamability of the surfactant solution [117]. Dynamic surface tension depends on many factors such as CMC, surfactant structure, surfactant concentration, temperature, electrolyte, micellar stability or relaxation time (τ_2_), *etc.* [116,118,119,120,121]. From the micellar stability point of view, stable micelles with a longer τ_2_ result in higher dynamic surface tension with a low surface tension gradient and lower foamability, because stable micelles cannot provide a high enough number of surfactant molecules to the newly created interface [122,123].

Foam stability is defined as the collapse time to reach 50% of the original foam height. Factors affecting the stability are the surface elasticity, viscosity (bulk and surface), gravity, and capillary suction [124]. Drainage is induced by gravitational force and capillary suction and can be decreased by increasing the bulk viscosity and the surface dilational elasticity. Bulk viscosity is known to delay the thinning of thick water channels. It is also noted that the surface viscosity and surface elasticity can be improved by increasing the surface adhesion of surfactant layers, enabling the film to adjust against stress and reducing drainage [124]. The ideal foaming solution would exhibit the highest level of foam stability as well as a minimum level of organelle destabilization. Lastly, for practical applications, easy scale-up production with a low cost would increase the feasibility of using the foaming materials.

#### 4.3.3. Status

**Figure 11:**
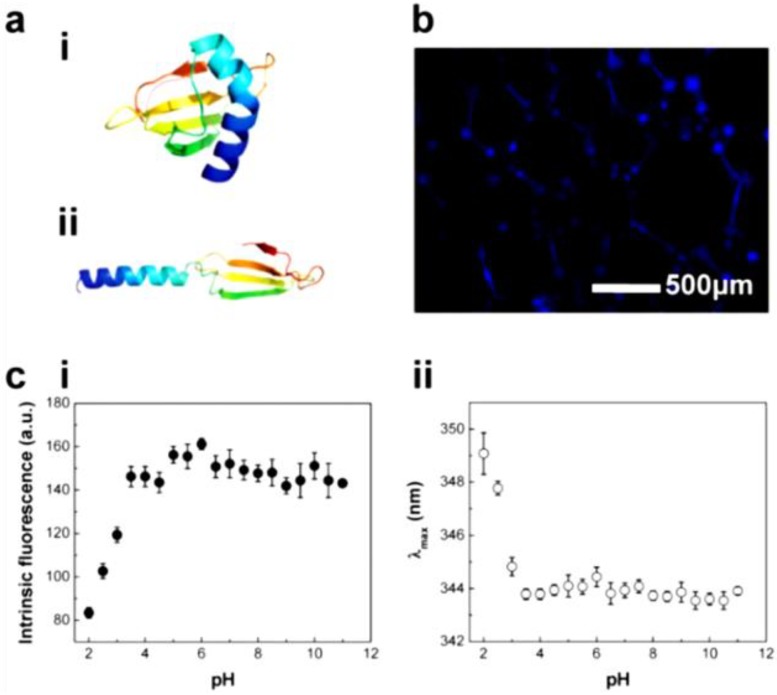

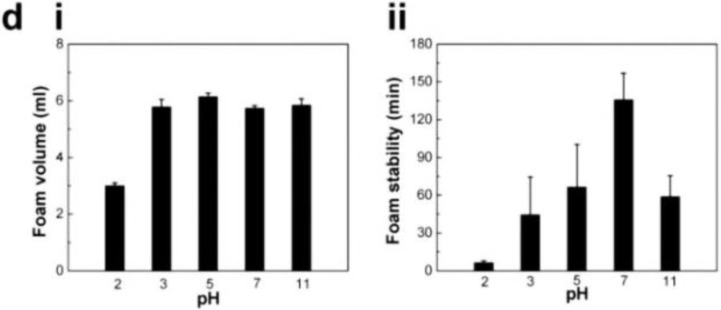
(**a**) Ranaspumin (RSN)-2 conformation: (**i**) globular and (**ii**) extended form (adapted with permission from [128]. Copyright 2010 Elsevier); (**b**) Fluorescence microscopy image of fluorescamine-labeled RSN-2 foam; (**c**) Intrinsic fluorescence of RSN-2 foams: (**i**) maximum emission intensity and (**ii**) maximum emission wavelength (λ_max_); (**d**) The effects of pH on the characteristics of RSN-2 foam: (**i**) foamability and (**ii**) foam stability. (**c**,**d**) reprinted with permission from [127]. (Copyright 2013 IOP Publishing)

**Figure 12:**
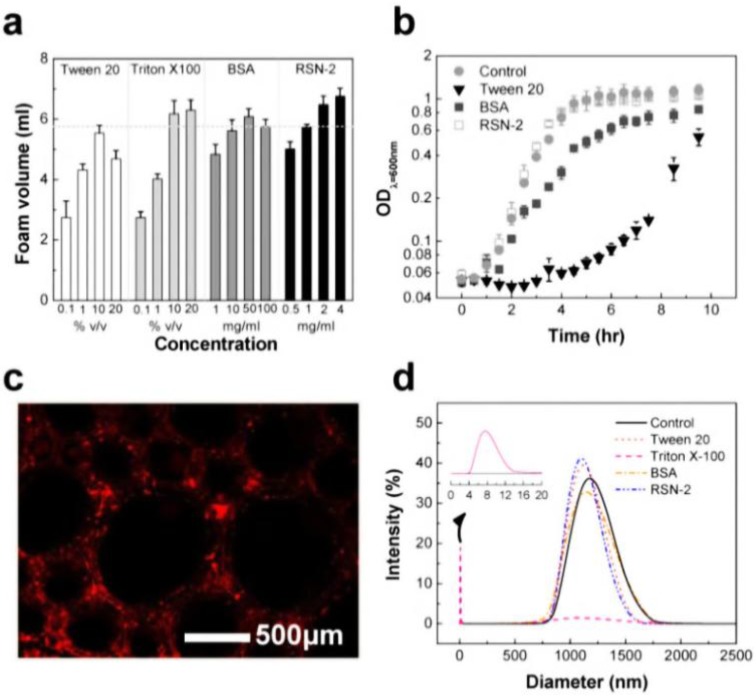
(**a**) The foamability of Tween 20, Triton X-100, BSA, and RSN2 foaming solutions. Biocompatibility of natural/artificial cellular systems: (**b**) bacterial *E. coli* cells; (**c**) sulforhodamine-encapsulating liposomes in RSN-2 foam; and (**d**) dynamic light scattering (DLS) spectra of liposomes collected from the drainage solution of the foam samples. Data reprinted with permission from [127]. Copyright 2013 IOP Publishing.

We paid attention to the naturally occurring frog foam system to study how it protects eggs from unfavorable environments and microbes. Kennedy and his colleagues found that six kinds of ranaspumin (RSN) proteins comprise the foam nests of the túngara frog [125]. RSN-2 is composed of a single α helix and a four-stranded β sheet. It exhibits strong surfactant performance through a folding-unfolding conformational change [126]. Upon mechanical agitation, a globular form of RSN-2 (Figure 11a(i)) turns into an extended conformation (Figure 11a(ii)), resulting in foaming (Figure 11b). An important aspect to note is that RSN-2 maintained its native tertiary structure at pH ≥ 3.5 as evidenced by intrinsic fluorescence intensity (Figure 11c(i)) and wavelength at maximum emission (Figure 11c(ii)). pH-dependent structural stability of the protein showed a good correlation with foamability, suggesting that foamability decreased with the degree of denaturation (Figure 11d(i)). The highest level of foam stability was observed at near neutral pH due to the absence of net charge (Figure 11d(ii)) [127]. An amount of 1 mg mL^−1^ of RSN-2 exhibited similar levels of foamability to highly concentrated surfactants (Tween 20, Triton X-100, and BSA), supporting excellent foamability of RSN-2 (Figure 12a). Most importantly, among all tested surfactants, RSN-2 exhibited the most excellent biological compatibility with natural organisms (see Figure 12b for *E. coli* cell growth, influenza virus stability) and liposomes (see Figure 12c for fluorescence microscope image of liposomes in RSN-2 foam and Figure 12d for dynamic light scattering of liposomes from foam drainage). Moreover, it is worth noting that RSN-2 expression in a bacterial system was used to ensure high throughput production of the protein. Overall, these experimental results promise the potential applicability of artificially reconstructed biofoams for the creation of *in vitro* biochemical reactor.

#### 4.3.4. Prospects

When we look into the future, foam architecture could be used as an alternative to conventional cell culture methods. The key advantage of using foam architecture for cell culture over conventional methods is the innate high surface area-to-volume ratio, which guarantees the best aeration. The oxygen transfer rate ($N_{0})$ is influenced by a specific surface area (*a*), which is a function of the surface area-to-volume ratio: $N_{0}=k_{L}a\cdot (C_{sat}-C)$, where $C_{sat}$ and C are oxygen concentrations of the gas phase in equilibrium with the saturated oxygen concentration and the actual oxygen concentration in the liquid, respectively, and *k_L_* is a mass transfer coefficient [129,130]. In the case of microbial culture, shaking or agitation provides sufficient oxygenation for respiration. However, mechanical agitation can be significantly reduced in the case of foams due to their large surface area-to-volume ratio. Further, culturing cells in thin aqueous channels may reduce risk of contamination by microorganisms, because protein surfactants layer acts as a protective barrier against invasion of airborne biological/chemical contaminants into the suspension of cell and media. As a result, foam architecture may provide a sterile environment, which allows cellular metabolism to take place, and can be used for cell cultivation with high cell viability. Even though protein-based foam architecture is believed to ensure a safe biological/physical environment to culture cells with good quality and quantity, its application is currently limited due to the stability issue. Therefore, if stability requirements are met by the development of optimal foaming formulation, biofoams would provide a convenient and useful approach to cell culture biotechnology.

### 4.4. Biofuel Production & Carbon Dioxide Fixation

#### 4.4.1. Background

According to the statistical review of world energy in 2013, primary energy consumption increased by 1.8% in 2012 and oil remains the major primary fuel at 33.1% of global energy consumption [131]. Fossil fuels still represents the major energy source, accounting for a continued increase of CO_2_ emissions. It is predicted that by 2030, fossil fuels will play a major role in meeting the global energy supply and renewable energy including biofuels will grow fast (7.6%) to meet about 17% of the global energy demand [132]. One interesting aspect to note is that from 2011 to 2030, as a result of the penetration of renewable energy into the market of global energy production, the role of coal and gas will be reduced. Another noteworthy aspect is that CO_2_ emissions will increase by 26% during the same period mainly due to the combustion of fossil fuel, which is a concern for environmental protection and global health. Overall, there is a need for the development of new fuel technology to satisfy the requirements of both efficient energy production and CO_2_ mitigation [133,134].

Consequently, biofuel production from biomass such as food crops and microalgae has been extensively investigated as a potential candidate to meet the aforementioned technical challenges. However, energy production using crop plants such as sugar cane, wheat, soybean, palm, and corn has raised ethical problems because more than half of the human population are malnourished and 52.5% of child deaths worldwide are associated with undernutrition [135,136]. Other problems are soil erosion/contamination (fertilizer, insecticides, and herbicides), extensive use of water (15 L per 1 L of ethanol), carbon dioxide generation due to the use of fossil energy, and the large land area needed.

In the case of cellulosic materials, a larger amount of biomass is required to produce the same amount of sugars or starches compared to corn, and cellulose degradation technology using biological (enzymatic digestion) and chemical process (heat/acid treatment) must be better developed for both large-scale application and reduction in production cost [136,137,138]. Microalgal biofuel production has gained a lot of attention due to the fact that its production does not rely on soil fertility, and cells are cultured in closed bioreactors or in open ponds. In addition to the advantage of ease of scale up, since microalgae consume CO_2_ during growth, CO_2_ in the exhaust gas from factories could be used to culture cells. As a result of its high oil content, microalgae biofuel is considered to be very strong candidate as an alternative to diesel fuel [139]. However, because of low light-to-biomass conversion efficiency and low biomass production, various research activities have been directed toward the reactor design, process (growth, environmental conditions, harvest, biofuel conversion) optimization, and engineering (reduction of antenna size) of algae to drive photosynthesis efficiently and thus to increase biomass (see [140,141,142,143,144,145,146] for reviews). Development of technologies to narrow the gap between theoretical estimation and real production would be a determining factor for microalgal biofuel to maintain competitive advantage.

Based on the background mentioned above, a photosynthetic system was replicated *in vitro* by mimicking the Calvin cycle in plant cells for sugar synthesis and carbon fixation [133] (for reviews on photosynthesis, see [147,148,149,150,151,152]). For this purpose, aforementioned ATP-producing vesicles and the Calvin cycle enzymes were encapsulated in a foam bioreactor. ATP could then be used by the Calvin cycle enzymes to produce glyceraldehyde-3-phosphate (G3P), which can be converted to glucose. Interestingly, BR-ATP synthase-vesicles yielded higher photosynthetic activity in RSN-2 foams than in bulk solution. As a result, a higher level of chemical conversion efficiency (~96%) was estimated in the foam compared to the bulk solution (~53%); the photosynthetic foam exhibited 116 nmol of glucose/(mL/h). Using a few assumptions, the photosynthetic foam was calculated to have ten times higher dimethylfuran (DMF) productivity than that from other types of biomass [133]. Unlike conventional biomass technology, a foam-based bioreactor does not require extensive investment of resources such as land, water, and energy to harvest sugars. Therefore, it is anticipated that a foam-based photosynthetic system combined with an artificial cellular system may provide another route for future biofuel production.

#### 4.4.2. Mechanism and Design Parameters

**Figure 13:**
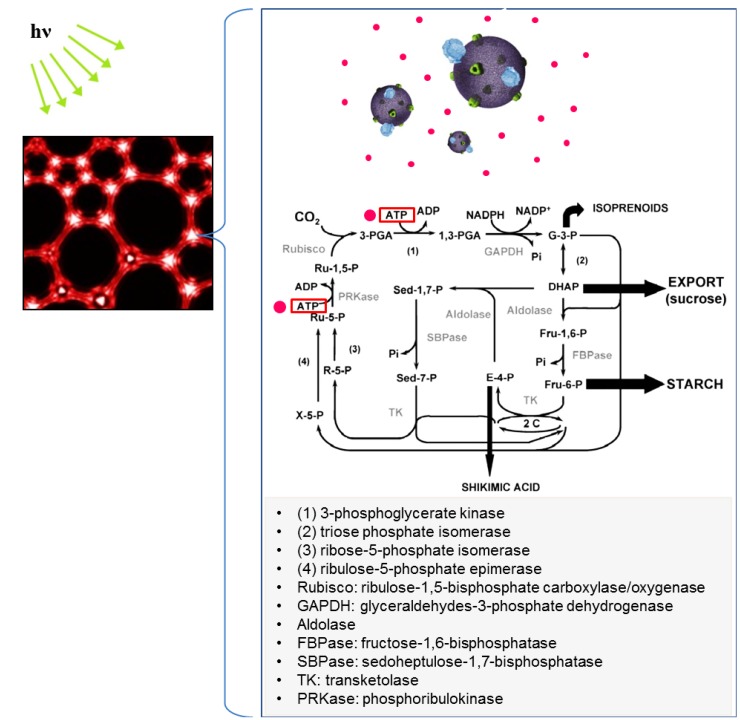
Application of ATP-producing polymersomes to replicating the artificial Calvin cycle in the foam to produce biofuels and carbon fixation. The Calvin cycle diagram is adapted with permission from [152]. Copyright 2003 Springer Science and Business Media.

**Figure 14:**
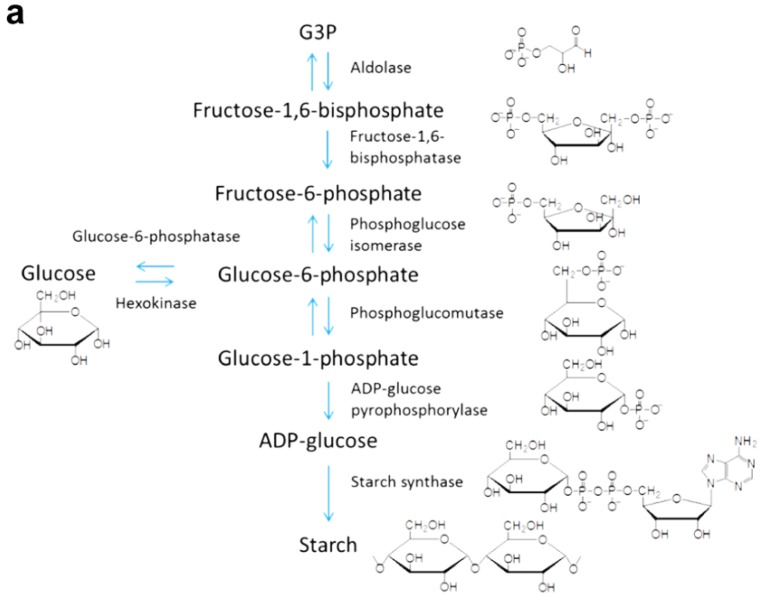

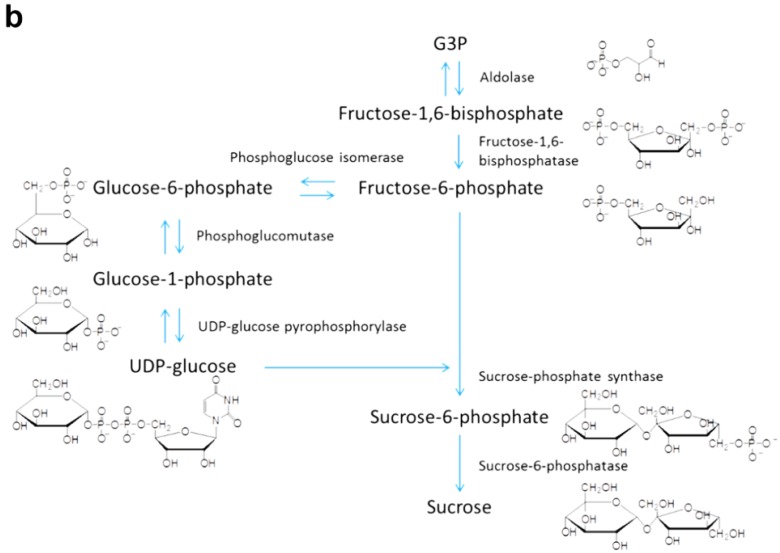
The conversion process of glyceraldehyde-3-phosphate (G3P) to (**a**) starch； and (**b**) sucrose. Intermediates and enzymes are shown at each reaction step.

As shown in Figure 13, carbon dioxide fixation is catalyzed by a 5-carbon ribulose biphosphate carboxylate (Rubisco), forming a 6-carbon molecule [153]. This molecule splits into two phosphoglycerate (PGA), which are reduced to form G3P; G3P molecules are used to synthesize carbohydrates such as fructose, glucose, sucrose, and starch, which can be converted to biofuel (conversion methods are reviewed in [154,155,156,157,158]). Metabolic pathways for G3P to starch and G3P to sucrose are shown in Figure 14a,b, respectively.

Although early studies utilized the steps involved in carbon fixation and reduction of 3-phosphoglycerate (3-PGA) to G3P in the Calvin cycle, all Calvin cycle enzymes and ATP-producing systems may lead to continuous metabolic reactions such as carbon fixation and carbohydrate production under controlled conditions. A critical point to consider is that the accumulation or withdrawal of intermediates such as G3P in the bioreactor will result in the decrease of available phosphate needed for ATP synthesis in the BR-ATP synthase-polymersomes, leading to reduction of ATP supply for the Calvin cycle. In plants, the G3P/phosphate translocator in the chloroplast envelope enables the continuous production of ATP through the regulatory exchange of G3P in the chloroplast with the phosphate in the cytosol [159]. This exchange cannot take place in the *in vitro* system shown in Figure 13. Therefore, it may be necessary to supplement the bioreactor with inorganic phosphate so as not to impede the photosynthetic Calvin cycle. Moreover, the oxidation of NADPH molecules provides reducing power for the production of G3P in the Calvin cycle and NADP^+^ is then reduced by electron transport chains in the thylakoid membrane [160,161,162]. In the artificial Calvin cycle system, polymersomes serve a similar function to the thylakoid membrane in terms of ATP production. Thus, one of the future challenges is to devise the NADP^+^ to NADPH reduction method in the polymersome-based artificial Calvin cycle. Ferredoxin-NADP reductase involved in photosynthesis may be used as a key element to reduce NADP^+^ [163,164,165,166]. Also, the function of Calvin cycle enzymes depends on various biological, chemical, and physical factors. For example, the factors affecting the overall carboxylation activity of Rubisco include 1) active conformational change by carbamylation at basic pH and stabilization with Mg^2+^, 2) competitive reaction between CO_2_ and O_2_, 3) temperature stability, and 4) conversion to active conformation by ATP-dependent Rubisco activase [153,167,168]. Since multiple enzymes/biochemical reactions, polymersomes, and environmental factors (e.g., CO_2_, temperature, pH, light incubation) are involved in replicating the Calvin cycle, another important task will be to optimize material/process parameters and thus, to gain control over the working conditions for the maximum level of photosynthesis.

#### 4.4.3. Prospects

Reconstruction of the Calvin cycle *in vitro* strongly depends on the optimal ATP production from artificial organelles (light dependent reaction) and selection/regulation of highly active enzymes for high photosynthetic efficiency (light independent reaction). The relationship between enzyme activity and Calvin cycle flux is reviewed by Raines [152] and here we summarize briefly the main features of essential enzymes described therein. In terms of enzyme activity, the low photosynthesis flux control coefficients (*C*^J^ = *d*(lnJ)/*d*(lnE), J and E are flux and enzyme activity, respectively) of GAPDH (<0.2), FBPase (<0.2), and PRKase (<0.28) indicates that the change in the activity of the enzyme had a negligible effect on the control of the Calvin cycle. In contrast, the response of Aldolase (*C*^J^: 0.07–0.55), TK (*C*^J^: 0.07–1.0), and SBPase (*C*^J^: 0.3–0.75) turned out to exert significant control over the Calvin cycle. Rubisco revealed a wide range of variation in *C*^J^ (0–1) depending on the growing and measuring conditions, however, in general, photosynthesis was insensitive to the decrease in Rubisco activity with *C*^J^ < 0.2. Although regulations of Calvin cycle enzymes are not fully understood, we draw our attention to the fact that SBPase has a significant impact on photosynthetic carbon fixation and biomass in transgenic plants [169,170,171,172,173,174]. That is, the possibility of controlling the Calvin cycle by modulating one or a few enzymes and/or sugar signaling mechanisms will save us a considerable amount of engineering effort in applying the principle of the plant life process to solving environmental problems. In addition, it is predicted that selection and modification of optimal enzymes, finding *in vitro* parameters affecting enzyme activity, regulation of enzyme activity, design of the most efficient photosynthetic pathways using key enzymes, and controllability of individual process in the pathway will be critical to develop optimally functioning systems.

## 5. Conclusions

Biomimetic membrane technology offers a unique opportunity to use transmembrane proteins for the development of engineering devices. As such, the development of a protein-embedded polymer membrane must meet several biological and engineering requirements. First, proteins must preserve their original functionality in the artificial membrane. Since proteins comprise active component of the system, it is critical to optimize the process conditions and material properties, including polymers, for the highest level of protein activity. Second, the polymer should exhibit the following characteristics: (i) mechanical/chemical/long-term stability; (ii) chemically functionalizable structure; (iii) controlled quality; and (iv) easy-to-scale up—(i), (iii), and (iv) are required for industrial applications. The potential advantage of using polymers as building blocks of biomimetic membrane comes from the diverse possible modifications of the polymer and functionalization (ii). Such polymers can be used to make mechanically stronger membranes, form networks with surrounding materials, establish intravesicular networks, and adjust the biochemical stability of the membranes, which has been impossible with natural lipids. As a result, polymer-based biomimetic membranes offer significant benefits over natural membranes.

Although they are still at an early stage of development, protein-incorporated polymer membranes have already been used in solving current energy and environmental problems by both academia and industry. This is important evidence to show that ideas from natural biological systems can provide a number of useful methods to solve technical problems. As summarized in this review, Aqp-incorporated polymer membranes (polymersomes, planar membranes) could be applied to develop RO membranes. In addition, it was demonstrated that ATP-producing polymersomes could be used to replicate the artificial Calvin cycle in the biofoams for biofuel production/carbon dioxide sequestration. However, in spite of significant progress, it appears unlikely that they are close to the level of commercialization, owing largely to lower performance or higher cost than conventional systems. This further implies that much more work still needs to be done to maintain competitiveness against the existing technology. In turn, the competitive position of the biomimetic membrane technology, as has been predicted by the life-like performance of proteins, can be achieved through optimal design of materials and devices. We believe that understanding the fundamental characteristics of biomimetic polymers would provide application-specific design strategies for the development of water purification membranes using Aqp-incorporated polymer membranes and biofuel production/carbon sequestration using foam-based bioreactors.
